# Comparison of Three Bed Bug Management Strategies in a Low-Income Apartment Building

**DOI:** 10.3390/insects3020402

**Published:** 2012-04-02

**Authors:** Changlu Wang, Kurt Saltzmann, Gary Bennett, Timothy Gibb

**Affiliations:** 1 Department of Entomology, Rutgers University, New Brunswick, NJ 08901, USA; 2 Department of Entomology, Purdue University, West Lafayette, IN 47907, USA; E-Mails: saltzman@purdue.edu (K.S.); gbennett@purdue.edu (G.B.); gibbs@purdue.edu (T.G.)

**Keywords:** *Cimex lectularius*, infestation, integrated pest management

## Abstract

Bed bug (*Cimex lectularius* L.) infestations are currently controlled by a variety of non-chemical and chemical methods. There have been few studies on the comparative effectiveness of these control techniques. We evaluated three bed bug management strategies in an apartment building: (1) non-chemical methods only (n = 9); (2) insecticides only (n = 6); and (3) integrated pest management including both non-chemical methods and insecticides (n = 9). The apartments were one-bedroom units occupied by seniors or people with disabilities. Bed bug numbers in each apartment were determined by visual inspection and/or installing intercepting devices under bed and sofa legs. The median (min, max) bed bug counts in the non-chemical methods only, insecticides only, and integrated pest management (IPM) treatment were: 4 (1, 57), 19 (1, 250), and 14 (1, 219), respectively prior to the treatments. The apartments were retreated if found necessary during biweekly to monthly inspections. After 10 weeks, bed bugs were found to be eliminated from 67, 33, and 44% of the apartments in the three treatment groups, respectively. The final (after 10 weeks) median (min, max) bed bug counts in the non-chemical methods only, insecticides only, and IPM treatment were: 0 (0, 134), 11.5 (0, 58), and 1 (0, 38), respectively. There were no significant differences in the speed of bed bug count reduction or the final bed bug counts. Lack of resident cooperation partially contributed to the failure in eliminating bed bugs from some of the apartments. Results of this study suggest that non-chemical methods can effectively eliminate bed bugs in lightly infested apartments.

## 1. Introduction

Bed bug (*Cimex lectularius* L*.*) infestations have increased rapidly in recent years throughout the United States as well as in Canada, Eastern Europe, Australia, and some African and Asian countries [1,2,3,4,5,6]. Bed bug infestations can rapidly expand once introduced into a multi-occupancy building [7]. The use of insecticides is commonly recommended for complete eradication of bed bugs [8]. Widespread bed bug resistance to commonly used insecticides (pyrethroids) in the U.S. is becoming evident [9,10]. In addition, applying insecticides directly to mattresses or upholstered furniture creates a high risk of human-pesticide exposure. Therefore, there has been significant interest in using non-chemical methods and an integrated pest management (IPM) approach to eliminate bed bugs.

Many different non-chemical tools and techniques designed for bed bug control are available. Some of the common tools/techniques include mattress encasements, hot steam, containerized heat treatment, whole house heat treatment, laundering, and freezing [11,12,13]. Wang *et al*. (2009) [7] demonstrated the effectiveness of two IPM strategies incorporating hot steam, encasements, intercepting devices, and frequent laundering in apartments. Until present, data on the comparative effectiveness of non-chemical control strategies are lacking. The objective of this study is to compare three bed bug treatment strategies in a low-income apartment building. Our hypotheses are that non-chemical tools alone can eliminate bed bugs in lightly infested apartments and IPM will achieve faster control than using insecticides alone. 

## 2. Methods

### 2.1. Study Site

We conducted the study in a 15-story apartment building managed by the Indianapolis Housing Agency. The building has 223, one-bedroom, apartments occupied by low-income, elderly or disabled tenants. Over 100 apartments had bed bug infestations as of February 2008. Educational brochures and videos were distributed or shown to residents between 2009 and 2010 by Purdue University researchers. An experienced member of the housing complex staff applied hot steam to kill bed bugs when requested by residents or when new infestations were found during maintenance work. Monthly pest control service was provided by a professional pest control contractor.

We obtained a list of apartments that were reported to have bed bug infestations. These units and their adjacent apartments received a thorough visual inspection and/or monitoring by installing Climbup^TM^ Insect Interceptors (Susan McKnight, Inc, Memphis, TN) under bed and sofa legs for two weeks. Twenty four apartments were identified as infested and were used for this May to August 2010 study. Initial bed bug counts ranged from 1 to 250. These counts were based on interceptor and/or visual inspection counts. An educational brochure about bed bug biology and control was provided to all participating residents.

### 2.2. Treatment Methods

The bed bug-infested apartments were divided into three treatment groups. Each group received one of the following treatments: (1) non-chemical methods only (n = 9); (2) insecticides only (n = 6); and (3) IPM including both non-chemical and chemical methods (n = 9). The median (min, max) bed bug counts in the non-chemical methods only, insecticides only, and IPM treatments were: 4 (1, 57), 19 (1, 250), and 14 (1, 219), respectively. True traditional controls (no treatments applied) were not feasible in this study due to the expectation of residents with bed bugs and the moral obligation of the researchers to provide some control efforts. Our previous experience had indicated that the use of non-chemical methods only cannot effectively eliminate bed bugs from heavily infested apartments (*i.e.*, >100 bed bugs) within an acceptable period of time. Therefore, we did not assign heavily infested apartments to the non-chemical only treatment and assigned more lightly infested (*i.e.*, with < 10 bed bugs) units in this group.

Otherwise, the apartments were randomly assigned to one of the three treatments and were treated immediately after the initial counts were obtained. The non-chemical treatment included installing encasements to mattresses and box springs, applying hot steam using a Ladybug XL2300 (Advanced Vapor Technologies, Edmonds, WA), and hand removal of bed bugs using forceps while conducting inspections. No insecticides were applied in this group. The IPM treatment included all of the non-chemical tactics above plus applying 0.075% Temprid SC (imidacloprid and cyfluthrin) (Bayer Environmental Science, Research Triangle Park, NC) spray or Tempo dust (1% cyfluthrin) (Bayer Environmental Science) or Mother Earth D (diatomaceous earth) (BASF Corporation, St. Louis, MO) dust to cracks or seams of bed frames and sofas and perimeter of the sleeping areas. Saltzmann and Wang performed the non-chemical and IPM treatments, except hot steam application, which was provided by a trained, housing staff member. The time (mean ± SEM) spent for the initial treatment in the non-chemical and IPM groups was 21 ± 7, and 26 ±7 minutes, respectively. The insecticides only treatment included the use of Temprid spray by the pest control contractor, and an additional treatment of Tempo or Mother Earth D dust by the researchers. The pest control contractor applied Temprid spray in the insecticide only group during their normal monthly pest control visit. Their spray treatment might not be very thorough based on the short service time (a few minutes per apartment) logged on their treatment report. Researchers only applied insecticide dust to harborages where bed bugs were found or likely to be present. In all apartments, interceptors were placed under all bed and sofa legs to monitor bed bug numbers. Residents in all treatment groups were asked to launder their clothing and bed linens regularly and eliminate “bridges” between furniture and the walls or floor. Bed bugs can use such bridges to circumvent interceptor devices placed under furniture legs. In reality, however, few of the tenants fully followed these recommendations. 

After the initial treatments, the interceptors were inspected biweekly to monthly by Saltzmann, a technician, and/or Wang until 30 November 2010 or until zero bed bugs were found after two consecutive monitoring checks. The interceptors were wiped clean then re-lubricated with talcum powder at each visit. A brief visual inspection also was conducted if time allowed and the rooms were accessible. Additional treatments were applied when needed in the non-chemical methods only and IPM groups. Researchers informed the contractor of the bed bug counts after each interceptor trap assessment, which helped the contractor in targeting their treatments. In addition to the contractor’s service, researchers applied diatomaceous earth dust or Tempo dust to bed bug harborages during visual inspections in the insecticides only treatment group. This was necessary because each apartment was only visited once per month by the contractor and live bed bugs were found during our post-treatment inspection trips. 

Resident interviews were conducted in January 2011, approximately 5–8 months after initiating the treatments. Residents were asked the following three questions: (1) Are you still experiencing bed bug infestations? (2) How do you rate the bed bug control in the building? and (3) What steps have you taken to control bed bugs? In addition, interceptors were installed under bed and sofa legs for 14 days and a visual inspection was conducted to quantify bed bug population levels.

### 2.3. Data Collection and Analysis

The bed bug counts from interceptor catches and/or visual inspections were logarithmic transformed prior to analysis. The wk 0 transformed data were subject to Analysis of Variance to compare among treatments. The wk 0 to wk 10 transformed data were analyzed using the Proc Mixed procedure in SAS software to determine whether there were significant differences in the speed and level of bed bug reduction among the three treatments [14]. Data after wk 10 were not compared between treatments due to irregular monitoring and treatment schedules.

## 3. Results

The median (min, max) bed bug counts prior to treatment in the non-chemical, insecticides only, and IPM treatment groups were: 4 (1, 57), 19 (1, 250), and 14 (1, 219), respectively (Table 1). The final (after 10 weeks) median (min, max) bed bug counts in the non-chemical methods only, insecticides only, and IPM treatment were: 0 (0, 134), 11.5 (0, 58), and 1 (0, 38), respectively. The mean number of treatments received in each group was 2.5, 2.5, and 2.6, respectively, based on those apartments with ≥7 bed bugs at wk 0. After 10 weeks, bed bugs were eliminated from 67, 33, and 44% of the apartments in the three treatment groups, respectively. Use of interceptor traps also may have helped control the bed bug infestations by killing the trapped bed bugs. In the non-chemical methods only treatment, bed bugs were eliminated from 6 of the 7 apartments that had 1–12 bed bugs at wk 0. The other apartment became bed bug free after wk 12. The non-chemical strategy failed to eradicate bed bugs from two apartments that had 28 and 57 bed bugs at wk 0. Residents in both of these two apartments did not wash bed linens regularly and had their beds touching the walls during the study period. The insecticides only treatment resulted in the lowest elimination rate of the three strategies.

**Figure 1:**
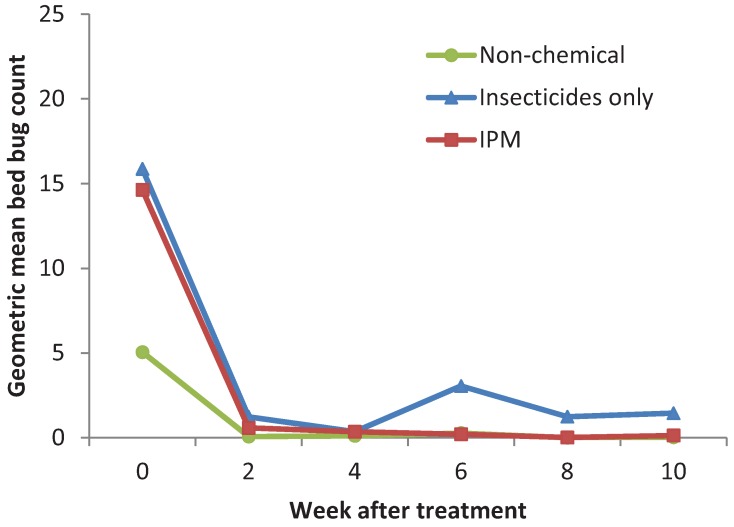
Effectiveness of three bed bug management strategies on bed bug populations.

Apartments with ≥7 bed bugs were used to compare the bed bug count reduction among the three treatments. There were no significant differences among treatments in the counts at wk 0 (F = 1.01; df = 2, 9; P = 0.40). The geometric means of bed bug counts in the non-chemical (n = 4), insecticides only (n = 4), and IPM (n = 5) treatments were 5, 18, and 23, respectively. After 10 wks, the geometric means reduced to 0, 0, and 2, respectively. The geometric mean bed bug count reduction in the three treatments was: 98.6, 61.2, and 99.9%, respectively. There were no significant differences in the speed of bed bug count reduction (F = 1.03; df = 11, 18; P = 0.46) or the final counts (F = 0.32; df = 2, 8; P = 0.73) among the treatments (Figure 1).

**Table 1:** Effectiveness of three bed bug treatment strategies after 10 weeks.

Treatment	Median (min, max) bed bug count at wk 0	Number of apartments with bed bugs eliminated/total number of apartments		
		With <10 bed bugs at wk 0	With >10 bed bugs at wk 0	Total
Non-chemical	4 (1, 57)	5/6	1/3	6/9
Insecticides only	19 (1, 250)	2/2	0/4	2/6
IPM	14 (1, 219)	1/4	3/5	4/9

In January 2011, 12 test apartments (three non-chemical methods only, six insecticides only, and three in the IPM treatment group) were reexamined to determine the long-term effectiveness of the treatments. Five of them (two in non-chemical methods only, two in insecticides only, and one in IPM treatment) still had bed bug infestations based on interceptor catches and visual inspections. These apartments remained continuously infested between the initial treatment date and 30 November 2010. The rest of the test apartments were not surveyed due to lack of access, vacancy, or turnover of tenants. Residents in three of the five infested apartments reported that they were aware of the bed bug infestations in their units. Nine of the 10 residents interviewed rated the bed bug control service “good” or “very good”. One resident rated the service as “fair”. Among the 10 residents surveyed, 100% used non-chemical methods to control bed bugs. Only one resident personally used chemicals for controlling bed bugs during the study period.

## 4. Discussion

We demonstrated that resident education, employment of simple non-chemical tools/techniques, and periodic bed bug monitoring and trapping can effectively eradicate light bed bug infestations. Although the process was slow, this non-chemical strategy is safe to the environment and human health and can be easily done with minimal training. These findings highlight the importance of educating the public about bed bugs, proactive monitoring, and early interventions. All of these help in effectively, safely, and economically eradicating bed bug infestations. Education, identification and control of bed bugs in their early stage are most important in low-income communities, where people often cannot afford more expensive, professional services.

Each of the three treatment groups still had two apartments with >10 bed bugs after 10 weeks. The IPM treatment did not provide faster control than the other two treatments as we hypothesized. A variety of factors resulted in the slow reduction of bed bugs. We partially relied on the residents, property management staff, and the existing pest control service provider for treatment implementation with the hope that the bed bug management program would be sustainable after the withdrawal of the researchers. Although interviews seemed to show that residents were cooperating, many residents did not fully follow the researchers’ instructions (frequent laundering, de-cluttering, eliminating bridges between beds and walls and floors). The bed legs in some rooms could not fit into interceptors or the mattresses were not on bed frames. One heavily infested sofa always had bed bugs. It was difficult for hot steam to penetrate to the space behind the thick cushions of the sofa. Some apartments were not accessible during biweekly to monthly monitoring trips because residents did not want to be bothered. The maintenance staff was not always competent in identifying bed bugs by visual inspection. The pest control contractor’s monthly service was very cursory (only a few minutes per apartment) based on their service report. As reported from a previous study in the same building, this contractor’s service was sometimes only able to suppress the bed bug populations, not being able to provide bed bug elimination in all cases [15]. All these factors negatively influenced the effectiveness of the treatments. Researchers applied diatomaceous earth or Tempo dust in the insecticides only treatment group during visual inspections when necessary. Without these additional treatments, the bed bug numbers in this treatment group might be higher at the end the study. 

One of the original research objectives was to compare the cost and pesticide use of the insecticide only and IPM treatments. It is expected that including non-chemical methods such as applying hot steam, installing encasements, discarding heavily infested materials will reduce the number of chemical applications or quantity of insecticides that is needed. However, bed bug infestation levels in the building became much lower by the time this project was initiated. The small numbers of samples and large variances made it impossible to compare the amount of pesticide used among the treatments. Further studies using larger sample sizes are necessary to determine the differences in cost and pesticide use of various treatment strategies. Establishing these parameters will help communities, as well as pest control providers, in selecting the most cost-effective means to manage bed bug infestations.

Lack of efficacy of available insecticides for bed bugs is considered as a major factor responsible for the bed bug control difficulties [16]. The lack of efficacy of insecticide treatments is partly due to the widespread bed bug resistance to pyrethroid insecticides [9,10]. It often requires multiple visits and weeks of time to eliminate an infestation using pyrethroid insecticides [17]. Similar to the two previous bed bug control studies in the same building conducted by our group [7,17], the various treatments were able to reduce bed bugs to very low levels after 8 weeks, but were not able to eradicate bed bugs in all test units. Laboratory assays using bed bugs collected from the building showed the bed bugs were readily killed by pyrethroid direct sprays at label rates (Wang, unpublished data). But continuous exposure to dry Suspend SC (0.038% deltamethrin) residues for 24 h only resulted in approximately 50% mortality (Wang, unpublished data). The results further confirm the importance of precise application when using insecticide sprays in bed bug control to achieve satisfactory results. Careful inspections would be required in each home visit to identify the bed bug hiding places. A more effective alternative to using insecticide sprays is to use dust formulations, which are more effective than spray formulations [18]. Bed bug legs and body have abundant setae that easily pick up dry dust when they crawl over treated surfaces. However, applying insecticide dusts is potentially more dangerous to occupants and the dusts are often visible after applications. 

Bed bugs spread within a building through both active and human-mediated passive dispersal [15]. Education of residents helps reduce the probability of human-mediated passive dispersal, but cannot stop bed bugs from active dispersal because numerous potential dispersal pathways exist between apartments within a building. It is likely that the presence of low number of bed bugs in some test apartments at the end of the study was due to dispersal from neighboring infested units and/or passive dispersal. Conversely, the slow elimination process in the test apartments might have resulted in new infestations in the adjoining units. Thus, conducting building-wide intervention is important for bed bug management in multi-unit dwellings.

The failure of bed bug eradication in some apartments was always associated with a lack of cooperation from residents or very high levels of infestation at the beginning of the experiments. The lack of cooperation is often due to physical disabilities, financial difficulties of the residents, or indifference concerning bed bug infestations from residents. As long as these elements exist, managing bed bug infestations in low-income communities will continue to be a very challenging task. Future success of building-wide bed bug eradication in these communities will be dependent upon resident’s motivation, the level of assistance provided from building management staff, and competency of pest control providers.

## 5. Conclusions

Bed bugs can be effectively eradicated by economical non-chemical control methods when their infestation levels are low. The non-chemical methods only, insecticides only, and IPM treatments resulted in similar levels of bed bug control after 10 weeks. Regardless of the treatment strategies used, success in bed bug reduction and eradication are closely related to resident cooperation and thoroughness of treatment by the service providers.
